# Goldfish Response to Chronic Hypoxia: Mitochondrial Respiration, Fuel Preference and Energy Metabolism

**DOI:** 10.3390/metabo11030187

**Published:** 2021-03-22

**Authors:** Elie Farhat, Hang Cheng, Caroline Romestaing, Matthew Pamenter, Jean-Michel Weber

**Affiliations:** 1 Biology Department, University of Ottawa, Ottawa, ON K1N 6N5, Canada; efarh086@uottawa.ca (E.F.); hchen188@uottawa.ca (H.C.); caroline.romestaing@univ-lyon1.fr (C.R.); mpamenter@uottawa.ca (M.P.); 2 Univ Lyon, Université Claude Bernard Lyon1, CNRS, ENTPE, UMR 5023, LEHNA, F 69622 Villeurbanne, France; 3 Faculty of Medicine, University of Ottawa Brain and Mind Research Institute, University of Ottawa, Ottawa, ON K1H 8M5, Canada

**Keywords:** hypoxia tolerance, metabolic suppression, mitochondria, Na^+^/K^+^-ATPase, glycolysis, beta-oxidation, citrate synthase

## Abstract

Hypometabolism is a hallmark strategy of hypoxia tolerance. To identify potential mechanisms of metabolic suppression, we have used the goldfish to quantify the effects of chronically low oxygen (4 weeks; 10% air saturation) on mitochondrial respiration capacity and fuel preference. The responses of key enzymes from glycolysis, β-oxidation and the tricarboxylic acid (TCA) cycle, and Na^+^/K^+^-ATPase were also monitored in various tissues of this champion of hypoxia tolerance. Results show that mitochondrial respiration of individual tissues depends on oxygen availability as well as metabolic fuel oxidized. All the respiration parameters measured in this study (LEAK, OXPHOS, Respiratory Control Ratio, CCCP-uncoupled, and COX) are affected by hypoxia, at least for one of the metabolic fuels. However, no common pattern of changes in respiration states is observed across tissues, except for the general downregulation of COX that may help metabolic suppression. Hypoxia causes the brain to switch from carbohydrates to lipids, with no clear fuel preference in other tissues. It also downregulates brain Na^+^/K^+^-ATPase (40%) and causes widespread tissue-specific effects on glycolysis and beta-oxidation. This study shows that hypoxia-acclimated goldfish mainly promote metabolic suppression by adjusting the glycolytic supply of pyruvate, reducing brain Na^+^/K^+^-ATPase, and downregulating COX, most likely decreasing mitochondrial density.

## 1. Introduction

Hypoxia is a state of oxygen limitation commonly found in many environments. It presents a dangerous challenge requiring animals to enter a hypometabolic state for survival [1]. Under normoxic conditions, adenosine triphosphate (ATP) is mainly produced by oxidative phosphorylation (OXPHOS) in the mitochondria through the electron transport chain (ETC) [2,3]. In hypoxia-sensitive species, this critical pathway of energy metabolism is inhibited when O_2_ is scarce, causing an imbalance between the now lower supply of ATP and normal energetic demand [4]. By contrast, hypoxia-tolerant animals can maintain this balance by regulating the activity of key enzymes of energy metabolism (e.g., slowing the tricarboxylic acid (TCA) cycle and stimulating glycolysis) [3,5], and by downregulating ion pumps such as Na^+^/K^+^-ATPase [6]. Mitochondria are thought to play a key role in coordinating these responses because of their ability to detect changes in O_2_ [7]. Mitochondrial respiration can be affected by hypoxia acclimation differently, depending on metabolic fuel, species, and tissue. For instance, respiration capacity is decreased in the tissues of shovelnose ray sharks [8], oysters [9], and frogs [10], but maintained in epaulette sharks [8], killifish [11], and snappers [12]. Goldfish are among the champions of hypoxia tolerance [1], particularly at low water temperatures, but how their mitochondria respond to prolonged hypoxia is presently unknown. 

Goldfish can suppress metabolic rate by 42–74% to cope with a lack of O_2_ [13,14,15] and they have larger glycogen stores than hypoxia-sensitive species [16]. This indicates a potentially higher capacity for glycolysis and increased reliance on carbohydrates during prolonged hypoxia [17]. It would be useful to establish whether hypoxic goldfish favor carbohydrates over lipids by quantifying the activities of key enzymes involved in glycolysis and β-oxidation, and by testing the metabolic fuel preference of individual tissues. Therefore, the main goal of this study was to determine how prolonged hypoxia affects the respiration capacity and fuel selection of mitochondria in different goldfish tissues. To complement this investigation of fuel preference and to help identify potential mechanisms of metabolic suppression, we have also measured key enzymes of energy metabolism involved in glycolysis, β-oxidation and the TCA cycle, as well as Na^+^/K^+^-ATPase in brain, liver, and white muscle. We hypothesized that hypoxia-acclimated goldfish will (i) favor carbohydrates over lipids, and (ii) decrease overall flux capacity for energy metabolism to promote metabolic suppression. We anticipated that changes both in mitochondrial respiration and in enzyme activities would reflect these responses, but more strongly so in a critical tissue like the brain.

## 2. Results 

### 2.1. Mitochondrial Respiration

#### 2.1.1. LEAK

**Figure 1:**
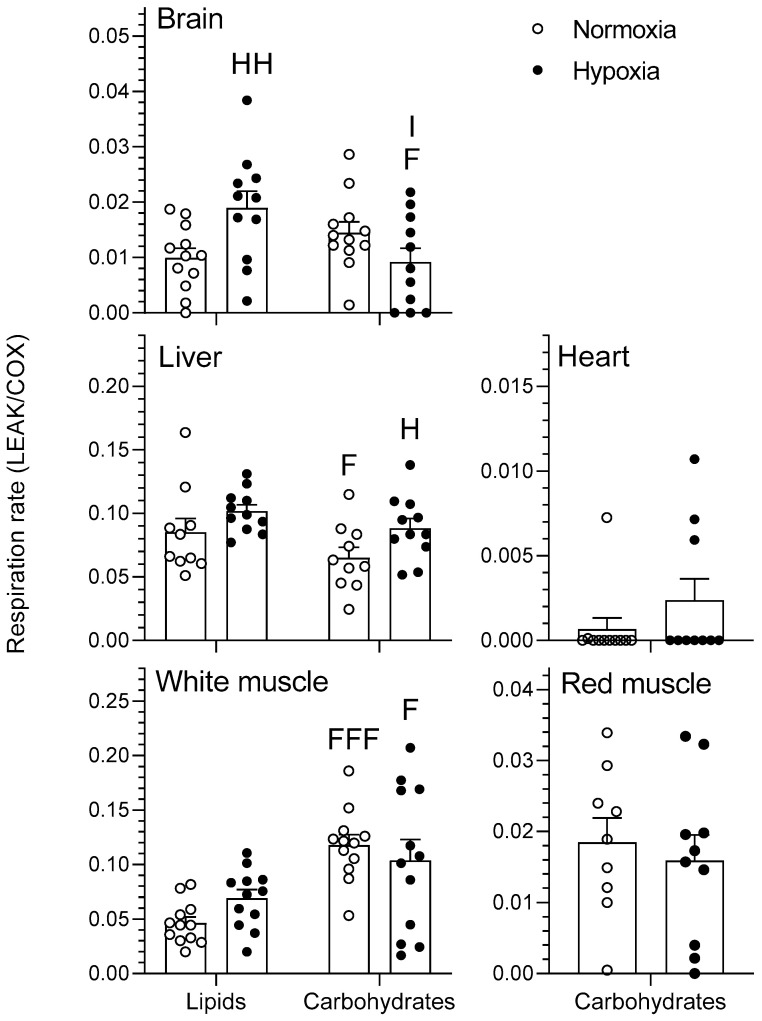
Mitochondrial oxidative fuel preference during nonphosphorylating respiration (LEAK) (pmol O_2_ sec^−1^ mg^−1^) normalized to cytochrome c oxidase (COX) respiration in the tissues of normoxic controls (*N* = 12) and hypoxia-acclimated goldfish (*N* = 11). LEAK represents mitochondrial respiration in the presence of pyruvate/malate for the carbohydrate protocol and palmitoylcarnitine/malate for the lipid protocol. Values are means ± standard error of the mean (s.e.m). Dots represent individual data points. Differences between fuels are indicated as F (*p* < 0.05) and FFF (*p* < 0.001). Differences between oxygen levels are indicated as H (*p* < 0.05) and HH (*p* < 0.01). Significant interaction between type of fuel and oxygen is indicated as I (*p* < 0.05).

The effects of chronic hypoxia and type of fuel on the respiration rate of mitochondria during nonphosphorylating respiration (LEAK) are presented in Figure 1. LEAK represents mitochondrial respiration in the presence of pyruvate/malate for the carbohydrate protocol and palmitoylcarnitine/malate for the lipid protocol. There was no effect of either O_2_ or fuel type on the heart or red muscle (*p* > 0.05). In normoxia, the liver consumed less O_2_ when using carbohydrates rather than lipids (*p* < 0.05). White muscle had higher O_2_ consumption when using carbohydrates than when using lipids for both normoxia (*p* < 0.001) and hypoxia (*p* < 0.05). Acclimation to hypoxia caused an increase in LEAK respiration of brain when using lipids and of liver when using carbohydrates (*p* < 0.05). Moreover, there was a significant interaction between the type of fuel used and chronic hypoxia acclimation in the brain (*p* < 0.05).

#### 2.1.2. OXPHOS 

**Figure 2:**
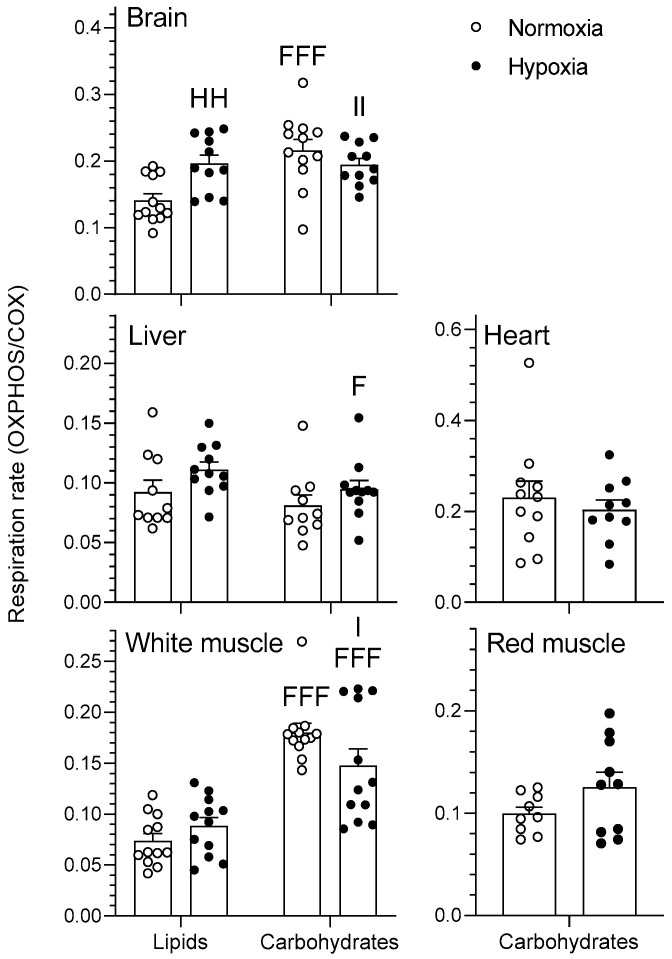
Mitochondrial oxidative fuel preference during oxidative phosphorylation (OXPHOS) respiration (pmol O_2_ sec^−1^ mg^−1^) normalized to COX respiration in the tissues of normoxic controls (*N* = 12) and hypoxia-acclimated goldfish (*N* = 11). OXPHOS represents mitochondrial respiration following the addition of saturating concentrations of ADP in the presence of pyruvate/malate for the carbohydrate protocol and palmitoylcarnitine/malate for the lipid protocol. Values are means ± s.e.m. Dots represent individual data points. Differences between fuels are indicated as F (*p* < 0.05) and FFF (*p* < 0.001). Difference between oxygen levels is indicated as HH (*p* < 0.01). Significant interactions between type of fuel and oxygen are indicated as I (*p* < 0.05) and II (*p* < 0.01).

**Figure 3:**
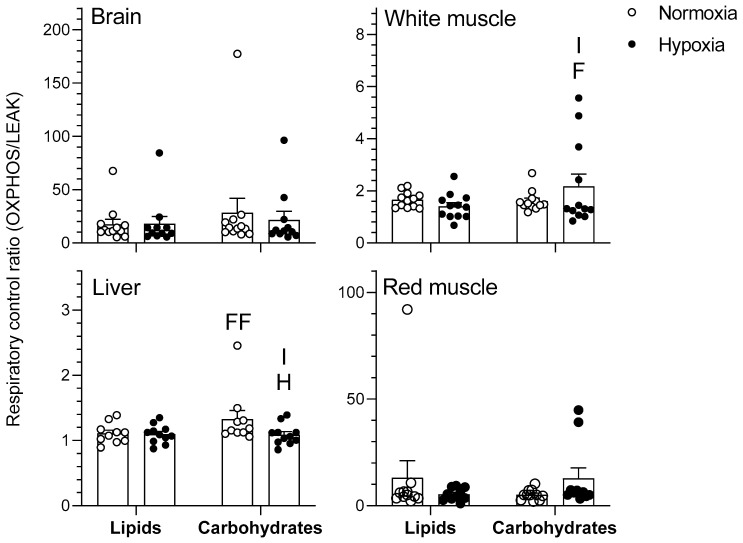
Respiratory control ratio (RCR) in the tissues of normoxic controls (*N* = 12) and hypoxia-acclimated goldfish (*N* = 11). RCR was calculated as the ratio between OXPHOS respiration and LEAK respiration (OXPHOS/LEAK). Values are means ± s.e.m. Dots represent individual data points. Differences between fuels are indicated as F (*p* < 0.05) and FF (*p* < 0.01). Difference between oxygen levels is indicated as H (*p* < 0.05). Significant interaction between type of fuel and oxygen is indicated as I (*p* < 0.05).

The effects of chronic hypoxia and type of fuel on OXPHOS respiration rate of mitochondria are presented in Figure 2. OXPHOS represents mitochondrial respiration following the addition of saturating concentrations of ADP in the presence of pyruvate/malate for the carbohydrate protocol and palmitoylcarnitine/malate for the lipid protocol. The type of fuel used had a significant effect on the OXPHOS respiration rates of goldfish mitochondria in various tissues. Specifically, respiration rates of normoxic brain as well as normoxic and hypoxic white muscle mitochondria were higher when using carbohydrates rather than lipids (*p* < 0.01). However, mitochondrial respiration rate of hypoxic liver was lower when using carbohydrates rather than lipids (*p* < 0.05). Neither heart nor red muscle OXPHOS respiration rates differed between oxygen levels (*p >* 0.05). Chronic hypoxia acclimation caused an increase in brain respiration rate when using lipids (*p* < 0.01) without affecting other tissues (*p >* 0.05). Additionally, there was an interaction between the two factors in both brain and white muscle that was only observed when normalizing to COX activity (*p* < 0.05). Mitochondrial respiratory control ratio (RCR), an index of mitochondrial coupling that was calculated as a ratio between OXPHOS and LEAK respiration, was higher in normoxic liver as well as hypoxic white muscle when using carbohydrates rather than lipids (*p* < 0.01). Chronic hypoxia caused a decrease in liver RCR when using carbohydrates (*p* < 0.05) without affecting other tissues (*p >* 0.05) (Figure 3). There was also a significant interaction between type of fuel and oxygen in liver and white muscle (*p* < 0.05). Finally, neither type of fuel nor hypoxia acclimation influenced brain or red muscle RCR (*p >* 0.05). OXPHOS respiration ranked in this order for lipids (heart > red muscle = brain > liver > white muscle) and this slightly different order for carbohydrates (heart > red muscle = brain > white muscle > liver) (*p* < 0.001) when not normalizing by COX (data not shown). For OXPHOS respiration normalized with COX (Figure 2), rates ranked in this order for lipids (brain > liver = white muscle) and for carbohydrates (heart = brain = white muscle > red muscle = liver) (*p* < 0.05).

#### 2.1.3. CCCP-Uncoupled State

**Figure 4:**
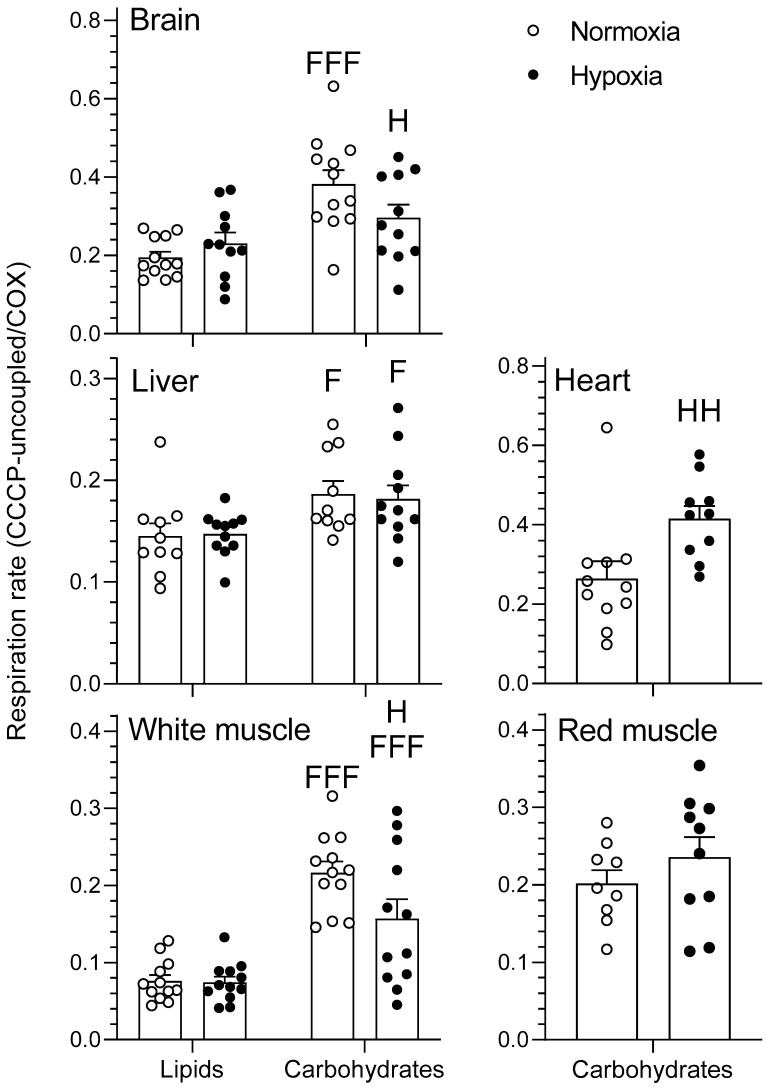
Mitochondrial oxidative fuel preference during the CCCP-uncoupled state (pmol O_2_ sec^−1^ mg^–1^) normalized to COX respiration in the tissues of normoxic controls (*N* = 12) and hypoxia-acclimated goldfish (*N* = 11). The CCCP-uncoupled state represents mitochondrial respiration following titration of carbonyl cyanide m-chlorophenyl hydrazone (CCCP) in the presence of pyruvate/malate + ADP + glutamate + succinate + cytochrome C for the carbohydrate protocol and palmitoylcarnitine/malate + ADP + cytochrome C for the lipid protocol. Values are means ± s.e.m. Dots represent individual data points. Differences between fuels are indicated as F (*p* < 0.05) and FFF (*p* < 0.001). Differences between oxygen levels are indicated as H (*p* < 0.05) and HH (*p* < 0.01).

The effects of chronic hypoxia and type of fuel on the respiration rate of mitochondria during the carbonyl cyanide m-chlorophenyl hydrazone (CCCP)-uncoupled state are presented in Figure 4. The CCCP-uncoupled state represents mitochondrial respiration following titration of carbonyl cyanide m-chlorophenyl hydrazone (CCCP) in the presence of pyruvate/malate + ADP + glutamate + succinate + cytochrome C for the carbohydrate protocol and palmitoylcarnitine/malate + ADP + cytochrome C for the lipid protocol. Respiration rate was higher when using carbohydrates rather than lipids in normoxic brain as well as in normoxic and hypoxic liver and white muscle (*p* < 0.05). Chronic acclimation to hypoxia caused a decrease in CCCP-uncoupled respiration rates of brain and white muscle, but an increase in heart, when using carbohydrates (*p* < 0.05). Finally, CCCP-uncoupled red muscle mitochondria were not affected by O_2_ (*p >* 0.05).

#### 2.1.4. Cytochrome Oxidase 

**Figure 5:**
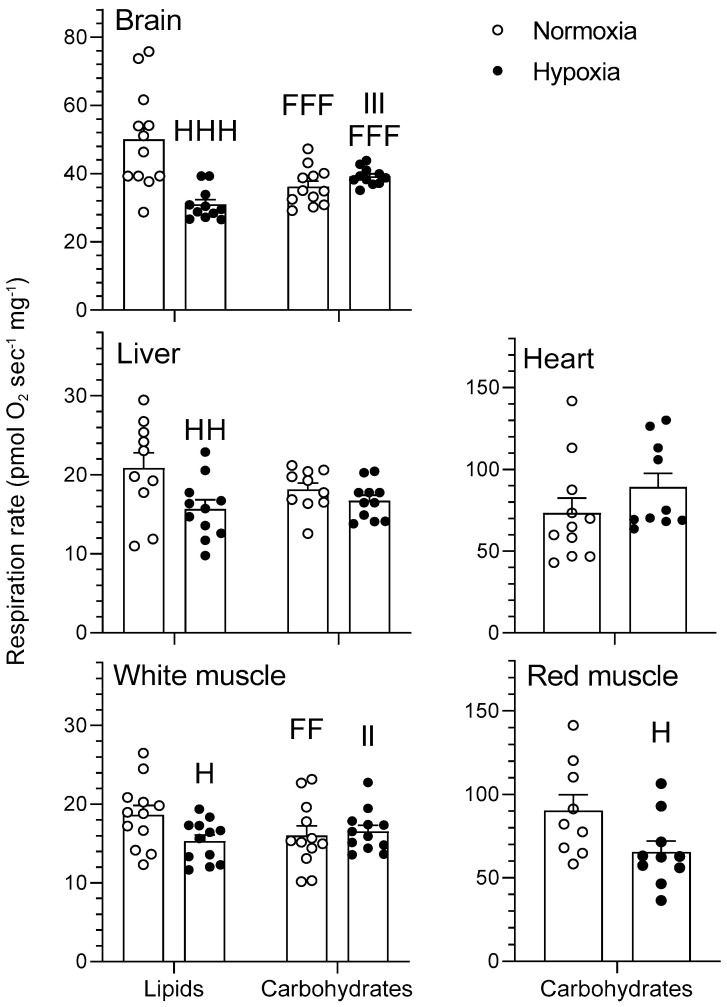
Mitochondrial oxidative fuel preference of COX in brain, liver, and white muscle of normoxic controls (*N* = 12) and hypoxia-acclimated goldfish (*N* = 11). COX respiration represents mitochondrial respiration following addition of ascorbate and a titration of N,N,N′,N′-tetramethyl-p-phenylenediamine (TMPD) in the presence of pyruvate/malate + ADP + glutamate + succinate + cytochrome c + CCCP + antimycin A for the carbohydrate protocol and palmitoylcarnitine/malate + ADP + cytochrome c + CCCP + antimycin A for the lipid protocol. Values are means ± s.e.m. Dots represent individual data points. Differences between fuels are indicated as FF (*p* < 0.01) and FFF (*p* < 0.001). Differences between oxygen levels are indicated as H (*p* < 0.05), HH (*p* < 0.01), and HHH (*p* < 0.001). Significant interactions between type of fuel and oxygen are indicated as II (*p* < 0.01) and III (*p* < 0.001).

The effects of chronic hypoxia and type of fuel on cytochrome c oxidase (COX) respiration are presented in Figure 5. COX represents mitochondrial respiration following addition of ascorbate and a titration of N,N,N′,N′-tetramethyl-p-phenylenediamine (TMPD) in the presence of pyruvate/malate + ADP + glutamate + succinate + cytochrome c + CCCP + antimycin A for the carbohydrate protocol and palmitoylcarnitine/malate + ADP + cytochrome c + CCCP + antimycin A for the lipid protocol. Both the type of fuel and O_2_ affected maximal COX respiration in goldfish brain and white muscle, but only O_2_ impacted the liver (Figure 5). Chronic hypoxia caused a decrease in COX respiration rate when using lipids in brain, liver, and white muscle (*p* < 0.05) as well as in red muscle (*p* < 0.05) when using carbohydrates, without affecting heart (*p >* 0.05). Moreover, COX respiration decreased in normoxic and hypoxic brain (*p* < 0.001) as well as normoxic white muscle (*p* < 0.01) when using carbohydrates rather than lipids (Figure 5). There were interactions between type of fuel and O_2_ in brain and white muscle COX. 

### 2.2. Energy Metabolism Enzymes

The effects of hypoxia acclimation on the activities of hexokinase (HK), pyruvate kinase (PK), lactate dehydrogenase (LDH), carnitine palmitoyl transferase (CPT), 3-hydroxyacyl CoA dehydrogenase (HOAD), and citrate synthase (CS) are shown in Table 1. The activity of HK increased in white muscle (82%; *p* < 0.01), decreased in brain (12%; *p* < 0.05), and was maintained in liver (*p >* 0.05). Moreover, chronic hypoxia caused a 47% decrease in liver PK activity (*p* < 0.05) as well as an 18% decrease in CPT and 70% increase in HOAD activity of brain (*p* < 0.05). Chronic hypoxia did not change the activities of LDH and CS in any measured tissue (*p >* 0.05).

### 2.3. Na^+^/K^+^-ATPase

**Figure 6:**
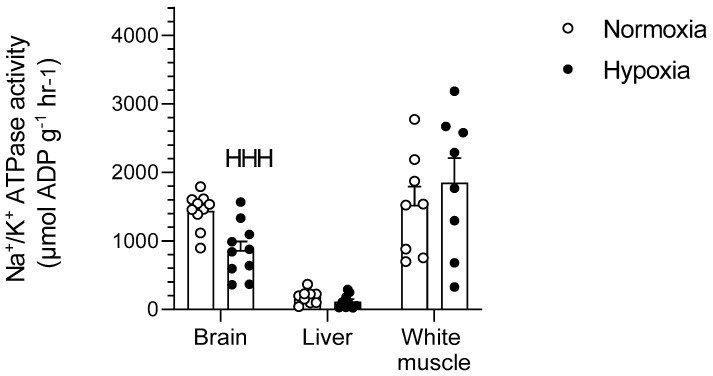
Effects of chronic hypoxia on Na^+^/K^+^-ATPase activity in the brain, liver, and white muscle of normoxic controls (*N* = 10) and hypoxia-acclimated goldfish (*N* = 10). Values are means ± s.e.m. Dots represent individual data points. Significant effect of hypoxia is indicated as HHH (*p* < 0.001).

Chronic hypoxia caused a 40% decrease in the activity of Na^+^/K^+^-ATPase in goldfish brains (*p* < 0.001) but did not affect liver or white muscle (*p >* 0.05) (Figure 6).

## 3. Discussion

This study is the first to investigate the effects of hypoxia acclimation on mitochondrial metabolism and Na^+^/K^+^-ATPase activity of the goldfish. It shows that the capacity for mitochondrial respiration of these champions of hypoxia tolerance depends on prevalent oxygen availability in the environment and on the type of metabolic fuel oxidized in a tissue-specific manner. OXPHOS respiration is higher for carbohydrates in brain and white muscle, whereas liver mitochondria reach higher maximal rates with lipids. Respiration rates are higher in brain when using lipids (LEAK and OXPHOS) and in liver when using carbohydrates (LEAK). Hypoxia acclimation causes significant tissue-specific changes in respiration rates of most tissues that differ with type of fuel and/or respiration state of mitochondria. COX respiration is lowered by chronic hypoxia acclimation in most tissues. In brain, hypoxia increases LEAK and OXPHOS respiration when using lipids, but decreases CCCP-uncoupled respiration when using carbohydrates. Therefore, hypoxia does not cause a consistent decrease in mitochondrial respiration capacity. Results also reveal that chronic hypoxia has widespread, tissue-specific effects on maximal flux capacity for glycolysis and β-oxidation, and causes strong downregulation of Na^+^/K^+^-ATPase in the brain. Hypoxia does not lead to universal tissue preference for carbohydrates over lipids. Instead, fuel selection strategies of individual tissues vary greatly. Overall, this study shows that hypoxia-acclimated goldfish mainly promote metabolic suppression by modulating glycolytic capacity and Na^+^/K^+^-ATPase, rather than by consistently downregulating mitochondrial respiration in all tissues.

### 3.1. Effects of Hypoxia on Mitochondrial Respiration 

Hypoxia has virtually no effects on LEAK, OXPHOS, and CCCP-uncoupled respiration when no normalization to COX is performed (results not shown). However, some significant changes in mitochondrial respiration capacity caused by acclimation to hypoxia become apparent when these respiration states are standardized per COX respiration (Figure 1, Figure 2, Figure 3 and Figure 4). This means that mitochondrial density is probably decreased by hypoxia in most tissues (see further discussion of COX respiration below). The effects of hypoxia acclimation differ depending on fuel type and mitochondrial respiration state. For example, brain respiration rates are increased by hypoxia during LEAK (substrates without ADP; complex I only; Figure 1) and OXPHOS (substrates with ADP; complexes I + II; Figure 2) (lipids; 39–91%) (Figure 1 and Figure 2), but lowered in the CCCP-uncoupled state (carbohydrates; 23%). Moreover, low O_2_ causes an increase in respiration rates of liver (carbohydrates; LEAK; 36%) and heart (carbohydrates; CCCP-uncoupled; 57%), but a decrease in liver (carbohydrates; RCR; 18%) and white muscle (carbohydrates; CCCP-uncoupled; 28%). After careful optimization of the liver and white muscle homogenates, we were still unable to reach high RCR values, indicating that preparation quality was lower. Reasons why this was the case are unknown, but respiration rates for these two tissues should be interpreted with caution. The decrease in liver RCR could mean that the mitochondria of this tissue are more uncoupled and less efficient because of the increase in LEAK and the lack of change in OXPHOS respiration. Results in muscle (white and red) and heart LEAK and OXPHOS are consistent with two previous studies on mitochondria from killifish liver [11] and snapper heart [12] that also maintain respiration capacity after acclimation. However, most ectotherms normally show a clear downregulation of mitochondrial performance when exposed to chronic hypoxia. They include shovelnose ray sharks [8], some mollusks [9], and frogs [10]. The absence of a clear downregulatory response of goldfish mitochondrial respiration in this study is unexpected because goldfish suppress whole-animal metabolic rate by 74% after 4 weeks at 10% air saturation [13]. General across-tissue changes in mitochondrial respiration capacity are unlikely to be involved in supporting such a deep hypometabolic state. It could be argued that selective pressure for mitochondrial plasticity to support hypoxia tolerance is lacking because the goldfish has evolved the capacity to survive in complete anoxia [18]. However, this reasoning can only be invoked at low temperatures because anoxic survival remains limited to less than a day at 20 °C and above [19]. As primary generator of cell power, mitochondria pump protons through their inner membrane via several enzyme complexes to establish an electrochemical gradient that eventually leads to the production of ATP at complex V [20]. Severe hypoxia depolarizes mitochondria, making complex V switch from producing ATP to consuming ATP [21]. This causes a mismatch between ATP supply and demand that affects tissue function. Complex IV (cytochrome c oxidase, COX) is a major contributor to the proton gradient as it uses O_2_, the final electron acceptor that is eventually reduced to water [22]. It is interesting to note that COX activity is reduced by hypoxia in most tissues. This enzyme complex is downregulated in brain, liver, white muscle, and red muscle following acclimation (Figure 5). It could indicate a possible regulation of the proton gradient in ways that decrease complex V activity, as observed in anoxic turtles that are also known for their extreme tolerance to hypoxia (in brain; [23], in heart, and liver; [24]). Reduced COX respiration could also mean that mitochondrial density is decreased by hypoxia acclimation in all these tissues (although the lack of change in CS activity does not support this notion; see Table 1). It is therefore possible that a reduction in COX activity could help metabolic suppression. Our study does not address the potential contribution of reactive oxygen species (ROS) to the hypoxia acclimation response. Investigating this contribution could be productive because the mitochondrion is a major producer of ROS, with the vast majority coming from complexes I and III [25]. This organelle is well positioned to sense any changes in O_2_ levels and initiate organism-specific adaptations. This sensing can be done acutely or chronically through an ROS-induced response that may cause rapid accumulation of Ca^2+^ and/or activation of hypoxia inducible factor (HIF) [20]. ROS can cause the formation of disulfide bonds, which may change the structure and function of proteins such as phosphatases, transcription factors, and those involved in epigenetic modifications [7]. Overall, the present results demonstrate that goldfish mitochondria respond in a tissue-specific manner to chronic hypoxia. Their response can differ between respiration states and is dependent on the type of fuel oxidized. Because goldfish mitochondria show no consistent decrease in respiration capacity, they appear to mainly support metabolic suppression by decreasing their number.

### 3.2. Tissue-Secific Fuel Preference of Goldfish Mitochondria 

Metabolic fuel preference of different goldfish tissues has not been investigated previously. Even for more thoroughly studied fish species like rainbow trout, quantitative information about substrate preference is scarce. Nevertheless, it is known that white muscle is mostly geared to use carbohydrates, whereas red muscle favors lipid oxidation [26]. Major indices of mitochondrial respiration (LEAK, OXPHOS, CCCP-uncoupled state, and RCR) are mostly dependent on the type of metabolic fuel oxidized. Furthermore, substrate preference shows great tissue specificity (Figure 1, Figure 2, Figure 3 and Figure 4). Respiration capacity is higher for carbohydrates than for lipids in the brain (normoxia; OXPHOS; 53%) and white muscle (normoxia and hypoxia; LEAK and OXPHOS; 39–154%), whereas it is the opposite in the brain (hypoxia; LEAK; 51%), and liver (normoxia and hypoxia; LEAK and OXPHOS; 12–36%; Figure 1 and Figure 2). Heart and red muscle of normoxic and hypoxic goldfish do not show preference for a particular fuel as they oxidize carbohydrates and lipids equally well. Overall results show interesting inter-tissue differences in fuel selection that are not affected by chronic hypoxia. 

In the CCCP-uncoupled state, mitochondrial respiration capacity is also higher in brain (normoxia and hypoxia; 28–96%), white muscle (normoxia and hypoxia; 111–184%), and liver (normoxia and hypoxia; 23–29%) when using carbohydrates rather than lipids (Figure 4). In normoxia and hypoxia, it is intriguing to see that the liver shows opposite fuel selection strategies between the CCCP-uncoupled state (Figure 4; carbohydrates > lipids) and LEAK and OXPHOS respiration (Figure 1 and Figure 2; lipids > carbohydrates). CCCP-uncoupled respiration only reflects substrate oxidation, independent of complex V, whereas OXPHOS is further constrained by ATP turnover [27]. The contrasting fuel preferences between CCCP-uncoupled and OXPHOS respiration of the liver indicate that mitochondrial capacity for carbohydrate oxidation is limited by maximal complex V activity in this tissue. The higher reliance of white muscle on carbohydrates (Figure 1 and Figure 2) is further supported by the higher mitochondrial RCR of this tissue when oxidizing carbohydrates (Figure 3). Overall, mitochondria favor the use of carbohydrates in brain and white muscle, prefer lipids in liver, and rely equally on both fuels in heart and red muscle. These tissue-specific patterns of fuel selection are largely independent of environmental oxygen availability.

### 3.3. Chronic Hypoxia and Glycolysis

Acclimation to low oxygen downregulates liver PK and brain HK, but upregulates white muscle HK (Table 1). Therefore, glycolytic capacity may be decreased in liver and brain, but increased in white muscle of hypoxia-acclimated goldfish. These tissue-specific responses show that aerobic supply of pyruvate from glycolysis to the TCA cycle varies between tissues. This indicates an increased reliance of white muscle on carbohydrates during chronic hypoxia and is also supported by (i) higher mitochondrial OXPHOS respiration (Figure 2), and (ii) higher glycogen stores in white muscle (but not liver) of goldfish acclimated to hypoxia [28]. The lack of change in LDH activity in hypoxia-acclimated goldfish suggests that anaerobic supply of ATP is not enhanced. This makes sense because goldfish do not rely on anaerobic ATP production at this level of hypoxia that does not cause the conversion of lactate to ethanol [13,29]. 

The variable glycolytic responses observed in different goldfish tissues do not allow to characterize a consistent pattern and echo the wide range of effects previously reported for ectotherms. For example, chronic hypoxia downregulates HK in tench white muscle [30], upregulates it in killifish brain [5], but maintains it in goldfish white muscle [31]. Variable responses have also been reported for liver PK, which is upregulated in Nile tilapia [32], but maintained in killifish [5] and in goldfish [31]. These different responses may be associated with various experimental conditions (e.g., temperature and diet) used in specific experiments on these different fish species. Under standardized conditions, it would be useful to explore whether glycolysis responds differently in hypoxia-tolerant vs. hypoxia-sensitive species, but no comparable data can be obtained for sensitive species because they cannot survive equivalent levels of hypoxia. Unfortunately, a common glycolytic response of hypoxia-tolerant ectotherms cannot be inferred from the current information. Like other species examined to date, the goldfish regulates the aerobic supply of ATP through glycolysis in a tissue-specific manner to cope with chronic hypoxia. 

### 3.4. β-Oxidation and TCA Cycle

Acclimation to low O_2_ causes no major changes in the capacity for β-oxidation and TCA cycle of goldfish tissues (Table 1). Aerobic supply of acetyl-CoA through these pathways is maintained during chronic hypoxia, even though demand is lowered by metabolic suppression [13]. This contrasts with previous studies on ectotherms that either downregulate [33,34,35] or upregulate [30,36] lipid catabolism and the TCA cycle. The only effects of chronic hypoxia on goldfish β-oxidation detected here were the downregulation of CPT and upregulation of HOAD in the brain (Table 1). The physiological implications of this contrasting brain response remain unclear, but results from brain mitochondria OXPHOS respiration (Figure 2) suggest an overall increase in β-oxidation capacity in this tissue during chronic hypoxia. 

### 3.5. Downregulation of Na^+^/K^+^-ATPase in Goldfish Brain 

The most striking physiological response to chronic hypoxia characterized here is a drastic downregulation of Na^+^/K^+^-ATPase in goldfish brain. The activity of this essential ion pump is decreased by 40% in the central nervous system, but remains unaffected in white muscle and liver (Figure 6). The same brain-specific response was previously reported in hypoxic naked mole-rats [37] and anoxic pond slider turtles [38]. However, it cannot be concluded that this physiological strategy is a required feature of the champions of hypoxia tolerance because crucian carp exposed to complete anoxia do not use it [38]. Most of the ATP consumed by the brain is used to maintain electrical activity by pumping ions [39]. Na^+^/K^+^-ATPase is arguably responsible for the majority of the brain ATP consumption and it is essential to maintain Na^+^ and K^+^ gradients, as well as to regulate Ca^2+^ and neurotransmitter transport [40]. Downregulating this pump will inevitably result in a decrease in O_2_ consumption. However, reducing Na^+^/K^+^-ATPase must occur concomitantly with a decrease in ion channel leak to maintain membrane gradients [41,42] and to protect against intracellular Ca^2+^ buildup that can lead to neuronal death [6]. A previous study shows that the anoxic goldfish brain downregulates Ca^2+^ channel activity together with Na^+^/K^+^-ATPase [43]. This suggests that channel arrest occurs when oxygen is absent, and determining whether it is also the case in chronic hypoxia will be a productive avenue for future research.

## 4. Methods

### 4.1. Animals

Adult common goldfish (*Carassius auratus* (Linnaeus 1758)) (*N* = 43, body mass 20.9 ± 0.2 g) were purchased from AQUAlity Tropical Fish Wholesale (Mississauga, ON, Canada) and held in a 1200 L flow-through holding tank in dechloraminated, well-oxygenated water, under a 12 h:12 h light:dark photoperiod, and were fed 3 mm floating fish pellets (Profishent; Martin Mills; Elmira, ON, Canada) once a day. They were randomly allocated to normoxia or hypoxia. All measurements were performed at 13 °C, and the fish were acclimated to this temperature for at least 2 weeks in the holding tank before starting experiments. Water was then made progressively hypoxic over 7 days by bubbling increasing amounts of N_2_ through a column filled with glass beads. Water PO_2_ was measured using galvanic oxygen probes (Loligo Systems, Tjele, Denmark). The probes were calibrated before each measurement using air-saturated water (20.9% O_2_). Oxygen availability went from 100% saturation on day 1 to 50, 40, 30, 20, 15, and finally 10% (or 2.1 kPa) on day 7. PO_2_ was maintained at that low level for a period of at least 4 weeks. This level of hypoxia was selected because it induces significant suppression of goldfish aerobic metabolism, but without causing any ATP synthesis from anaerobic ethanol production [13]. All procedures were approved by the Animal Care Committee of the University of Ottawa (protocol BL-1625) and adhered to the guidelines established by the Canadian Council on Animal Care for the use of animals in research.

### 4.2. Mitochondrial Respiration

After at least 4 weeks of acclimation to either normoxia or hypoxia, goldfish were quickly euthanized by cervical dislocation and brain, liver, white muscle, heart, and red muscle were carefully dissected. At least 150 mg of freshly collected brain, liver, and white muscle were quickly frozen in liquid N_2_ and stored at −80 °C for enzyme analyses. Approximately 30–50 mg of fresh brain, liver, white muscle, heart, and red muscle were placed in ice-cold BIOPS buffer (10 mM Ca-ethylene glycol-bis(β-aminoethyl ether)-N,N,N′,N′-tetraacetic acid (EGTA) buffer, 0.1 µM free calcium, 20 mM imidazole, 20 mM taurine, 50 mM 2-(N-Morpholino) ethanesulfonic acid potassium salt (K-MES), 0.5 mM 1,4-dithiothreitol (DTT), 6.56 mM MgCl_2_, 5.77 mM ATP, and 15 mM phosphocreatine, pH 7.1) for mitochondrial respiratory capacity measurements. 

Tissues were prepared for mitochondrial respiration in two different ways. Brain, liver, and white muscle were prepared by relying on a shredding technique adapted from [44,45,46,47,48]. Briefly, tissues were cut in ice-cold MiR05 (0.5 mM EGTA, 3 mM MgCl_2_.6 H_2_O, 60 mM lactobionic acid, 20 mM taurine, 10 mM KH_2_PO_4_, 20 mM 4-(2-Hydroxyethyl)piperazine-1-ethanesulfonic acid (HEPES), 110 mM D-sucrose, and 1 g L^−1^ fatty acid-free bovine serum albumin (BSA), pH 7.3) respirometry buffer using microdissecting scissors to obtain a small particulate solution (liver/white muscle) or by using a pestle for brain. The homogenization was completed by pipetting several times to obtain very small tissue pieces (tested by pipetting through a 1 mL tip for white muscle or 0.2 mL tip for brain and liver). The shredded homogenates were then diluted in MiR05 to obtain the desired final concentration (100 mg mL^−1^ for the liver, 60 mg mL^–1^ for the white muscle, and 5 mg mL^−1^ for brain). The entire procedure was carried out at 4 °C and completed within 20 min of the fish being euthanized. Red muscle and heart were used to prepare permeabilized muscle fiber bundles as previously described [49,50]. Briefly, a small piece of red muscle immersed in BIOPS was dissected to separate muscle fibers. Fiber bundles were transferred to BIOPS solution supplemented with saponin (50 μg mL^−1^) and mixed gently at 4 °C for 30 min. The permeabilized fibers were then gently washed once by mixing for 10 min at 4 °C in the Mir05 solution. Muscle fibers were then weighed, and their respiration was monitored with an Oroboros oxymeter at 13 °C in a hyperoxygenated respiratory buffer Mir05.

Mitochondrial respiration of all samples was measured at 13 °C using two Oxygraph-2k high-resolution respirometers (Oroboros Instruments, Innsbruck, Austria) running in parallel, each with a specific substrate protocol (carbohydrates or lipids). Oxygen concentration (pmol mL^−1^) was recorded using DatLab software (Oroboros Instruments). A two-point calibration of the oxygen electrodes was done daily: air saturation with the addition of oxygen and zero oxygen saturation with the addition of sodium dithionite. For the shredded preparation, homogenates were placed into the calibrated chamber with saponin (50 μg mL^−1^) for 15 min before starting any measurement. To avoid any limitation of oxygen diffusion to the cell, measurements were run under hyperoxygenated conditions (350–450 μmol L^−1^). Oxygen was added to the respirometry chambers at the beginning of the protocol. Mass-specific O_2_ consumption was expressed as pmol O_2_ s^−1^ mg^−1^ wet tissue. Two sequential substrate–uncoupler–inhibitor–titration (SUIT) protocols were run simultaneously for each tissue as follows: 

For the carbohydrate protocol, substrates were added in the following order: 5 mM/2.5 mM pyruvate/malate (PM) to ensure electron entry to complex I of the ETC (nonphosphorylating/LEAK), followed by saturating concentrations of ADP (1 mM for brain and 0.5 mM for liver/white muscle) to obtain the phosphorylating respiration rate/to activate ATP synthesis (OXPHOS), 10 mM glutamate to estimate amino acid utilization, 10 mM succinate to fully activate the ETC and obtain the real OXPHOS state by supporting electron entry to complex I + II, and 10 µM cytochrome C to test for the integrity of the outer mitochondrial membrane (rate below 15% was considered acceptable) [44]. A titration of 2 µM carbonyl cyanide m-chlorophenyl hydrazone (CCCP) was used to obtain the CCCP-uncoupled respiration rate, 2.5 µM antimycin A to inhibit complex III and obtain a residual oxygen consumption that is not linked to mitochondria. Finally, 2.5 µM ascorbate and a titration of N,N,N′,N′-tetramethyl-p-phenylenediamine (TMPD; final concentration = 2–2.5 mM TMPD) to measure complex IV state 3 respiration and obtain the maximal activity of cytochrome c oxidase (COX) as an estimate of mitochondrial density [51]. 

For the lipid protocol, substrates were added in the following order: 0.04 mM/2.5 mM palmitoylcarnitine/malate (PCM) to measure complex I + II respiration (nonphosphorylating/LEAK). At this concentration of malate, note that the exact contribution of complex II to the respiration rates measured is unclear. This is followed by the addition of a saturating concentration of ADP (as in the carbohydrate protocol), 10 µM cytochrome C, 2–4 µM CCCP, 2.5 µM antimycin A, 2.5 µM ascorbate, and finally, a titration of TMPD (final concentration = 2–2.5 mM; brain, liver, and white muscle only). Note that “carbohydrate protocol” and “lipid protocol” are so named to express what starting substrate is used in each procedure. COX activity was measured in both protocols in the presence of multiple substrates at the time antimycin A was added to the respirometer chamber.

### 4.3. Enzyme Assays

All enzyme activities were measured using a Spectra Max Plus384 Absorbance Microplate Reader (Molecular Devices, Sunnyvale, CA, USA). To measure the activities of key enzymes involved in (i) glycolysis (hexokinase (HK), pyruvate kinase (PK), and lactate dehydrogenase (LDH)), (ii) β-oxidation (carnitine palmitoyl transferase (CPT) and 3-hydroxyacyl CoA dehydrogenase (HOAD)), and (iii) the tricarboxylic acid cycle (citrate synthase (CS)), ~50 mg of each frozen tissue was weighed and homogenized on ice in 19 volumes of extraction medium (25 mM Tris/HCl + 1 mM EDTA as well as 5 mM dithiothreitol and 0.05% (*v*/*v*) Triton X-100 that were added on the day of the experiment to complete the enzyme extraction). Homogenates were then centrifuged at 4 °C at 2400× *g* for 5 min, and the resulting supernatant was stored at −80 °C until analyses. All homogenates were subjected to one freeze/thaw cycle. Preliminary experiments were carried out to ensure that all substrate and cofactor concentrations were saturating but not inhibitory. Control reactions (containing no substrate) were run simultaneously for each enzyme to measure background activity, if present. All assays were run in triplicate. 

Assay conditions were as follows: HK (A340; EC 2.7.1.1; [52]): 1 mM glucose, 5 mM MgCl_2_, 0.24 mM NADH, 2 mM phosphoenolpyruvate (PEP), 5 U/mL PK, 20 U/mL LDH, and 4 mM ATP (omitted from the control). PK: (A340; EC 2.7.1.40; [53]): 0.17 mM NADH, 5 mM ADP, 80 mM KCl, 10 mM MgCl_2_, 5 PEP (omitted from the control), and excess coupling enzyme (LDH) in 160 mM triethanolamine/HCl. LDH: (A340; EC; 1.1.1.27; [54]): 0.17 mM NADH, 1 mM KCN, and 2 mM pyruvate (omitted from the control) in 50 mM Tris/HCl. CS: (A412; EC 2.3.3.1; [55]): 0.2 mM 5,5′dithiobis(2-nitrobenzoic acid) (DTNB), 0.1 mM acetyl CoA, and 0.5 mM oxaloacetate (omitted from the control) in 50 mM Tris-HCl. CPT: (A412; EC 2.3.1.21; [56]): 0.15 mM DTNB, 0.035 mM palmitoyl CoA, carnitine (omitted from the control) in 50 mM Tris. HOAD: (A340; EC 1.1.1.35; [56]): 0.2 mM NADH, and 0.1 mM acetoacetyl CoA (omitted from the control) in 50 mM Imidazole + 1 mM EDTA.

We measured Na^+^/K^+^-ATPase activity (A340) by using a modified protocol from [57]. Frozen tissue was weighed (~100 mg) and homogenized on ice with a sonicator (Fisher Scientific Sonic Dismembrator model 100, San Diego, CA, USA) in a 4:1 SEI:SEID buffer (SEI: 250 mM sucrose, 10 mM EDTA, and 42 mM imidazole; SEID: 100 mL SEI + 0.5 g sodium deoxycholate). Homogenates were then centrifuged at 10,000× *g* for 5 min at 4 °C, and the resulting supernatant was directly used in the assay. The assay was performed as previously described [57] in quadruplicate (2 replicates contained 10 µL of homogenate + 200 µL of assay solution A (50 mM imidazole, 2.8 mM PEP, 0.7 mM ATP, 0.22 mM NADH, 5 mM PK, and 4 mM LDH) and 2 replicates contained 10 µL of homogenate + 200 µL of assay solution B (Solution A + 0.5 mM ouabain)). Ouabain was added to block Na^+^/K^+^-ATPase and measure any detectable ATP use not associated with this enzyme. 

### 4.4. Calculations and Statistics

Antimycin A values were subtracted from PM/PCM, ADP, glutamate, succinate, and CCCP values to obtain all desired states (LEAK, OXPHOS, and CCCP-uncoupled). Ascorbate values were subtracted from TMPD values to calculate COX activity. Mitochondrial respiratory control ratio (RCR), an indicator of mitochondrial coupling, was calculated as a ratio between OXPHOS and LEAK (OXPHOS/LEAK). All respiratory states were normalized to COX activity to avoid any intrinsic variation in mitochondrial density/content. Statistical analyses were performed using SigmaPlot 12.5 (Systat, San Jose, CA, USA). Data were analyzed using a repeated-measures two-way analysis of variance (RM-ANOVA) for the mitochondrial respiration experiments to test for significant interactions between the two independent variables: (i) oxygen level (normoxia vs. hypoxia) and (ii) type of fuel (lipids vs. carbohydrates), followed by the Holm–Sidak post hoc test. A two-tailed t-test was used to analyze the effects of hypoxia on all enzyme activities. Normality was assessed using the Shapiro–Wilk test, and homoscedasticity by the Levene’s test. When the assumptions of normality or equality of variances were not met, the data were normalized by log10. If transformation was unsuccessful, nonparametric Mann–Whitney U test was performed. All values presented are means ± standard error of the mean (s.e.m), and a level of significance of *p* < 0.05 was used in all tests.

## 5. Conclusions

This study shows that acclimation to hypoxia causes significant changes in mitochondrial respiration of all goldfish tissues. However, these mitochondrial responses vary greatly between tissues and often depend on the substrate being oxidized. All the respiration parameters measured here are stimulated or reduced by hypoxia, at least for one of the metabolic fuels tested: LEAK (brain and liver), OXPHOS (brain), RCR (liver), CCP-uncoupled (brain, white muscle and heart), and COX (all tissues except heart). Therefore, hypoxia acclimation causes a rather complex array of mitochondrial responses because no consistent across-tissue pattern could be established except for a general decrease in COX respiration. This observed change in COX activity suggests that hypoxia causes an organism-wide reduction in mitochondrial density. Downregulating COX could aid in achieving hypometabolism by indirectly inhibiting complex V via a reduction in the proton gradient. Regardless of environmental oxygen conditions, goldfish mitochondria favor the use of carbohydrates in brain and white muscle, prefer lipids in liver, and rely equally on both in heart and red muscle. Hypoxia causes the goldfish brain to switch to oxidizing lipids rather than carbohydrates with no clear preference observed in other tissues. Results also demonstrate a strong hypoxia-driven downregulation of brain Na^+^/K^+^-ATPase that supports whole-animal metabolic suppression and suggests concomitant channel arrest. The brain is the organ most affected by chronically low oxygen because it shows important responses in mitochondrial respiration (increase in LEAK and OXPHOS when using lipids, decrease in CCCP-uncoupled respiration when using carbohydrates, and decrease in COX when using lipids), as well as changes in the activities of key enzymes (HK, CPT, HOAD, and Na^+^/K^+^-ATPase). Overall, hypoxia-acclimated goldfish mainly promote metabolic suppression by regulating the glycolytic supply of pyruvate, downregulating brain Na^+^/K^+^-ATPase, and most likely decreasing mitochondrial abundance.

## Figures and Tables

**Table 1:** Effects of chronic hypoxia on the activities of key enzymes of glycolysis, β-oxidation and tricarboxylic acid (TCA) cycle in goldfish brain, liver, and white muscle (*N* = 10 for each treatment group). All values are given in µmol g^−1^ min^−1^ and presented as means ± s.e.m.

	Hexokinase		Pyruvate Kinase		Lactate Dehydrogenase		Carnitine Palmitoyl Transferase		3-Hydroxyacyl CoA Dehydrogenase		Citrate Synthase	
	Normoxia	Hypoxia	Normoxia	Hypoxia	Normoxia	Hypoxia	Normoxia	Hypoxia	Normoxia	Hypoxia	Normoxia	Hypoxia
Brain	16.64 ± 0.68	14.58 * ± 0.37	39.42 ± 3.06	37.75 ± 3.65	212.5 ± 13.23	204.12 ± 14.15	0.17 ± 0.01	0.14 * ± 0.04	0.1 ± 0.01	0.17 * ± 0.03	0.81 ± 0.24	0.57 ± 0.35
Liver	2.19 ± 0.23	1.89 ± 0.17	124.03 ± 20.95	65.15 * ± 14.38	344.89 ± 45.04	388.96 ± 43.38	10.34 ± 1.05	10.35 ± 1.04	0.29 ± 0.029	0.3 ± 0.03	2.09 ± 0.19	2.83 ± 0.42
White muscle	1.48 ± 0.11	2.69 ** ± 0.29	109.53 ± 5.2	96.63 ± 6.58	96.86 ± 13.51	118.53 ± 20.51	14.31 ± 1.67	11.73 ± 1.39	0.29 ± 0.05	0.46 ± 0.13	5.06 ± 0.31	4.71 ± 0.34

Differences between oxygen levels are indicated as * (*p* < 0.05) and ** (*p* < 0.01). Values in color show how hypoxia affects enzyme activity: green for activation and red for inhibition.

## Data Availability

The data presented in our study are openly available in Figshare at https://doi.org/10.6084/m9.figshare.14253224.v1 (accessed on 21 March 2021).

## References

[1] Bickler P.E., Buck L.T. (2007). Hypoxia tolerance in reptiles, amphibians, and fishes: Life with variable oxygen availability. Annu. Rev. Physiol..

[2] Semenza G.L. (2007). Life with oxygen. Science.

[3] Solaini G., Baracca A., Lenaz G., Sgarbi G. (2010). Hypoxia and mitochondrial oxidative metabolism. Biochim. Et Biophys. Acta (BBA)-Bioenerg..

[4] Boutilier R.G. (2001). Mechanisms of cell survival in hypoxia and hypothermia. J. Exp. Biol..

[5] Martínez M.L., Landry C., Boehm R., Manning S., Cheek A.O., Rees B.B. (2006). Effects of long-term hypoxia on enzymes of carbohydrate metabolism in the gulf killifish, fundulus grandis. J. Exp. Biol..

[6] Hochachka P.W. (1986). Defense strategies against hypoxia and hypothermia. Science.

[7] Pamenter M.E. (2014). Mitochondria: A multimodal hub of hypoxia tolerance. Can. J. Zool..

[8] Hickey A.J.R., Renshaw G.M.C., Speers-Roesch B., Richards J.G., Wang Y., Farrell A.P., Brauner C.J. (2012). A radical approach to beating hypoxia: Depressed free radical release from heart fibres of the hypoxia-tolerant epaulette shark (hemiscyllum ocellatum). J. Comp. Physiol. B.

[9] Sokolova I. (2018). Mitochondrial adaptations to variable environments and their role in animals’ stress tolerance. Integr. Comp. Biol..

[10] St-Pierre J., Tattersall G.J., Boutilier R.G. (2000). Metabolic depression and enhanced o2 affinity of mitochondria in hypoxic hypometabolism. Am. J. Physiol. Regul. Integr. Comp. Physiol..

[11] Du S.N., Mahalingam S., Borowiec B.G., Scott G.R. (2016). Mitochondrial physiology and reactive oxygen species production are altered by hypoxia acclimation in killifish (fundulus heteroclitus). J. Exp. Biol..

[12] Cook D.G., Iftikar F.I., Baker D.W., Hickey A.J., Herbert N.A. (2013). Low-o2 acclimation shifts the hypoxia avoidance behaviour of snapper (pagrus auratus) with only subtle changes in aerobic and anaerobic function. J. Exp. Biol..

[13] Farhat E., Turenne E.D., Choi K., Weber J.-M. (2019). Hypoxia-induced remodelling of goldfish membranes. Comp. Biochem. Physiol. Part B Biochem. Mol. Biol..

[14] Van Waversveld J., Addink A., van den Thillart G. (1989). The anaerobic energy metabolism of goldfish determined by simultaneous direct and indirect calorimetry during anoxia and hypoxia. J. Comp. Physiol. B.

[15] Van Ginneken V.J., Snelderwaard P., van der Linden R., van der Reijden N., van den Thillart G.E., Kramer K. (2004). Coupling of heart rate with metabolic depression in fish: A radiotelemetric and calorimetric study. Thermochim. Acta.

[16] Richards J.G. (2009). Metabolic and molecular responses of fish to hypoxia. Fish Physiology.

[17] Jibb L.A., Richards J.G. (2008). Amp-activated protein kinase activity during metabolic rate depression in the hypoxic goldfish, carassius auratus. J. Exp. Biol..

[18] Weber J.-M. (2016). Revealing how goldfish defy anoxia. J. Exp. Biol..

[19] Van den Thillart G., Berge-Henegouwen M., Kesbeke F. (1983). Anaerobic metabolism of goldfish, *Carassius auratus* (l.): Ethanol and CO_2_ excretion rates and anoxia tolerance at 20, 10, and 5 °C. Comp. Biochem. Physiol..

[20] McElroy G.S., Chandel N.S. (2017). Mitochondria control acute and chronic responses to hypoxia. Exp. Cell Res..

[21] St-Pierre J., Brand M.D., Boutilier R.G. (2000). Mitochondria as atp consumers: Cellular treason in anoxia. Proc. Natl. Acad. Sci. USA.

[22] Schmidt-Rohr K. (2020). Oxygen is the high-energy molecule powering complex multicellular life: Fundamental corrections to traditional bioenergetics. ACS Omega.

[23] Pamenter M.E., Gomez C.R., Richards J.G., Milsom W.K. (2016). Mitochondrial responses to prolonged anoxia in brain of red-eared slider turtles. Biol. Lett..

[24] Gomez C.R., Richards J.G. (2018). Mitochondrial responses to anoxia exposure in red eared sliders (trachemys scripta). Comp. Biochem. Physiol. Part B Biochem. Mol. Biol..

[25] Quinlan C.L., Perevoschikova I.V., Goncalves R.L., Hey-Mogensen M., Brand M.D. (2013). The determination and analysis of site-specific rates of mitochondrial reactive oxygen species production. Methods in Enzymology.

[26] Weber J.M., Haman F., Morris S., Vosloo A. (2004). Oxidative fuel selection: Adjusting mix and flux to stay alive. Animals and Environments.

[27] Bundgaard A., Qvortrup K., Rasmussen L.J., Fago A. (2019). Turtles maintain mitochondrial integrity but reduce mitochondrial respiratory capacity in the heart after cold acclimation and anoxia. J. Exp. Biol..

[28] van den Thillart G., Kesbeke F., van Waarde A. (1980). Anaerobic energy-metabolism of goldfish, *Carassius auratus* (l.). J. Comp. Physiol..

[29] Regan M.D., Gill I.S., Richards J.G. (2017). Calorespirometry reveals that goldfish prioritize aerobic metabolism over metabolic rate depression in all but near-anoxic environments. J. Exp. Biol..

[30] Johnston I.A., Bernard L.M. (1982). Ultrastructure and metabolism of skeletal muscle fibres in the tench: Effects of long-term acclimation to hypoxia. Cell Tissue Res..

[31] Van den Thillart G., Smit H. (1984). Carbohydrate metabolism of goldfish (*Carassius auratus* l.)–Effects of long-term hypoxia-acclimation on enzyme patterns of red muscle, white muscle and liver. J. Comp. Physiol. B.

[32] Mahfouz M.E., Hegazi M.M., El-Magd M.A., Kasem E.A. (2015). Metabolic and molecular responses in nile tilapia, oreochromis niloticus during short and prolonged hypoxia. Mar. Freshw. Behav. Physiol..

[33] Zhou B., Wu R., Randall D., Lam P., Ip Y., Chew S. (2000). Metabolic adjustments in the common carp during prolonged hypoxia. J. Fish Biol..

[34] Pillet M., Dupont-Prinet A., Chabot D., Tremblay R., Audet C. (2016). Effects of exposure to hypoxia on metabolic pathways in northern shrimp (pandalus borealis) and greenland halibut (reinhardtius hippoglossoides). J. Exp. Mar. Biol. Ecol..

[35] Li J., Xu X., Li W., Zhang X. (2018). Effects of acute and chronic hypoxia on the locomotion and enzyme of energy metabolism in chinese shrimp fenneropenaeus chinensis. Mar. Freshw. Behav. Physiol..

[36] Gerber L., Clow K.A., Katan T., Emam M., Leeuwis R.H., Parrish C.C., Gamperl A.K. (2019). Cardiac mitochondrial function, nitric oxide sensitivity and lipid composition following hypoxia acclimation in sablefish. J. Exp. Biol..

[37] Farhat E., Devereaux M.E.M., Pamenter M.E., Weber J.-M. (2020). Naked mole-rats suppress energy metabolism and modulate membrane cholesterol in chronic hypoxia. Am. J. Physiol. Regul. Integr. Comp. Physiol..

[38] Hylland P., Milton S., Pek M., Nilsson G.E., Lutz P.L. (1997). Brain na+/k+-atpase activity in two anoxia tolerant vertebrates: Crucian carp and freshwater turtle. Neurosci. Lett..

[39] Soengas J.L., Aldegunde M. (2002). Energy metabolism of fish brain. Comp. Biochem. Physiol. B.

[40] Erecińska M., Silver I.A. (1994). Ions and energy in mammalian brain. Prog. Neurobiol..

[41] Bickler P.E., Buck L.T. (1998). Adaptations of vertebrate neurons to hypoxia and anoxia: Maintaining critical ca2+ concentrations. J. Exp. Biol..

[42] Boutilier R., St-Pierre J. (2000). Surviving hypoxia without really dying. Comp. Biochem. Physiol. Part A Mol. Integr. Physiol..

[43] Wilkie M.P., Pamenter M.E., Alkabie S., Carapic D., Shin D.S.H., Buck L.T. (2008). Evidence of anoxia-induced channel arrest in the brain of the goldfish (carassius auratus). Comp. Biochem. Physiol. Part C Toxicol. Pharmacol..

[44] Kuznetsov A.V., Strobl D., Ruttmann E., Königsrainer A., Margreiter R., Gnaiger E. (2002). Evaluation of mitochondrial respiratory function in small biopsies of liver. Anal. Biochem..

[45] Kuznetsov A.V., Veksler V., Gellerich F.N., Saks V., Margreiter R., Kunz W.S. (2008). Analysis of mitochondrial function in situ in permeabilized muscle fibers, tissues and cells. Nat. Protoc..

[46] Salin K., Auer S.K., Rudolf A.M., Anderson G.J., Selman C., Metcalfe N.B. (2016). Variation in metabolic rate among individuals is related to tissue-specific differences in mitochondrial leak respiration. Physiol. Biochem. Zool..

[47] Larsen S., Kraunsøe R., Gram M., Gnaiger E., Helge J.W., Dela F. (2014). The best approach: Homogenization or manual permeabilization of human skeletal muscle fibers for respirometry?. Anal. Biochem..

[48] Velasco C., Draxl A., Wiethuchter A., Eigentler A., Gnaiger E. (2012). Mitochondrial respiration in permeabilized fibres versus homogenate from trout heart and liver. Mitochondrial Physiol. Netw..

[49] Pesta D., Gnaiger E. (2012). High-resolution respirometry: Oxphos protocols for human cells and permeabilized fibers from small biopsies of human muscle. Mitochondrial Bioenergetics.

[50] Bourguignon A., Rameau A., Toullec G., Romestaing C., Roussel D. (2017). Increased mitochondrial energy efficiency in skeletal muscle after long-term fasting: Its relevance to animal performance. J. Exp. Biol..

[51] Larsen S., Nielsen J., Hansen C.N., Nielsen L.B., Wibrand F., Stride N., Schroder H.D., Boushel R., Helge J.W., Dela F. (2012). Biomarkers of mitochondrial content in skeletal muscle of healthy young human subjects. J. Physiol..

[52] Best C., Melnyk-Lamont N., Gesto M., Vijayan M.M. (2014). Environmental levels of the antidepressant venlafaxine impact the metabolic capacity of rainbow trout. Aquat. Toxicol..

[53] Zammit V.A., Beis I., Newsholme E.A. (1978). Maximum activities and effects of fructose bisphosphate on pyruvate kinase from muscles of vertebrates and invertebrates in relation to the control of glycolysis. Biochem. J..

[54] Zammit V.A., Newsholme E.A. (1976). The maximum activities of hexokinase, phosphorylase, phosphofructokinase, glycerol phosphate dehydrogenases, lactate dehydrogenase, octopine dehydrogenase, phosphoenolpyruvate carboxykinase, nucleoside diphosphatekinase, glutamate-oxaloacetate transaminase and arginine kinase in relation to carbohydrate utilization in muscles from marine invertebrates. Biochem. J..

[55] Alp P.R., Newsholme E.A., Zammit V.A. (1976). Activities of citrate synthase and nad+-linked and nadp+-linked isocitrate dehydrogenase in muscle from vertebrates and invertebrates. Biochem. J..

[56] Guglielmo C.G., Haunerland N.H., Hochachka P.W., Williams T.D. (2002). Seasonal dynamics of flight muscle fatty acid binding protein and catabolic enzymes in a migratory shorebird. Am. J. Physiol..

[57] McCormick S.D. (1993). Methods for nonlethal gill biopsy and measurement of na+, k+-atpase activity. Can. J. Fish. Aquat. Sci..

